# Effects of perinatal asphyxia on cortical activity in two-year-old children

**DOI:** 10.1016/j.nicl.2025.103933

**Published:** 2025-12-17

**Authors:** Sebastian König, Anna Tuiskula, Marjo Metsäranta, Susanna Stjerna, Emma Saure, Leena Haataja, Sampsa Vanhatalo, Anton Tokariev

**Affiliations:** a Early Brain Activity, Systems and Health Group, University of Helsinki, Haartmaninkatu 8, 00290 Helsinki, Finland; b Department of Physiology, University of Helsinki, Haartmaninkatu 8, 00290 Helsinki, Finland; c Department of Neuroscience and Bioengineering, Aalto University, Rakentajanaukio 2, 02150 Espoo, Finland; d BABA Center, Pediatric Research Center, Clinical Trial Unit, Children’s Hospital of Helsinki University Hospital, Haartmaninkatu 8, 00290 Helsinki, Finland; e Department of Pediatrics, Children’s Hospital of Helsinki University Hospital, Stenbäckinkatu 9, 00290 Helsinki, Finland; f Division of Neuropsychology, HUS Neurocenter, Helsinki University Hospital and University of Helsinki, Haartmaninkatu 8, 00290 Helsinki, Finland; g Department of Pediatric Neurology, University of Helsinki and Helsinki University Hospital, Stenbäckinkatu 9, 00290 Helsinki, Finland; h Department of Clinical Neurophysiology, Children’s Hospital, HUS Imaging, Helsinki University Hospital, Stenbäckinkatu 9, 00290 Helsinki, Finland

**Keywords:** Electroencephalography, Neonate, Hypoxic-ischemic Encephalopathy, Perinatal Asphyxia, Connectivity

## Abstract

Perinatal asphyxia can lead to clinical hypoxic-ischemic encephalopathy (HIE) associated with high morbidity and mortality, but less is known about long-lasting effects of perinatal asphyxia alone (PA). Here, we investigate how PA with versus without clinical HIE affects cortical activity networks at two years of age. Electroencephalographic (EEG) recordings were acquired during sleep from three cohorts of children (PA only (n = 10), PA with mild to moderate HIE (n = 8), and healthy controls (n = 37)), and we assessed the group differences in local cortical function and cortico-cortical networks. Compared with the healthy controls, both PA and HIE were linked to reduced frequency-specific amplitudes. In two-year-old children with PA, the amplitude-related networks were stronger at low frequencies and weaker at higher frequencies, however, two-year-olds with HIE showed decreased connectivity at all frequencies. Likewise, phase-related networks in children with PA were stronger at lower frequencies and weaker at higher frequencies. Local phase-amplitude coupling was affected by PA or HIE in only a few cortical regions. Our findings suggest that PA, even without clinical HIE, may be associated with long-lasting changes to both local cortical activity and the large-scale cortical networks, which could potentially affect normal brain functions.

## Highlights

• Perinatal asphyxia may have a long-lasting effect on brain functions and cortical network activity
• Our findings suggest that perinatal asphyxia, even without clinical encephalopathy, reflects on cortical activity at age two
• Long-term effects of perinatal asphyxia on cortical activity networks appear to co-vary with neonatal encephalopathy severity

## 1 Introduction

Perinatal asphyxia, *i.e.*, insufficient blood flow and/or interrupted gas exchange to the foetus, can lead to hypoxic-ischemic encephalopathy (HIE). Globally, perinatal asphyxia is one of the most significant causes of neonatal mortality and morbidity (Lawn et al., 2005, Saugstad, 2011). Cognitive development is known to be affected when perinatal asphyxia leads to moderate or severe HIE (Grossmann et al., 2023, Lee and Glass, 2021). According to recent work, it appears that mild HIE also alters cognitive development (Finder et al., 2020). However, it remains unclear whether perinatal asphyxia without HIE (hereafter referred to as PA) could also have a fine-tuned effect on cognitive development (Finder et al., 2020, Tuiskula et al., 2025). Our recent work demonstrated that even PA or mild HIE influenced the neonatal cortical activity networks (Syvälahti et al., 2024), known to be associated with cognitive performance (Colonnese et al., 2010, [AuthorError] et al., 2019, Ahtola et al., 2023).

Electroencephalography (EEG) allows the non-invasive measurement of neural cortical activity and is widely used in clinics to assess early brain development. Multi-channel EEG can be readily used to estimate both local cortical function and large-scale interaction networks. Amplitudes of EEG signals reflect the synchronous co-activation of underlying neural populations. Cortical activity networks can be computed based on correlations of amplitude envelopes or phase synchronization between pairs of neural oscillations. Amplitude-amplitude correlations (AAC) are associated with the coordination of gross cortical activity at a multi-second timescale (Yrjölä et al., 2024, Hipp et al., 2012, Engel et al., 2013). In turn, phase-phase correlation (PPC) networks are thought to capture synchronization of neural peak excitability at the millisecond timescale (Galindo-Leon et al., 2019, Palva and Palva, 2012). It was shown that PPC networks are linked to early neurodevelopment (Yrjölä et al., 2022b, Tokariev et al., 2019b) and cognitive performance in adults (Hirvonen et al., 2018, Lobier et al., 2018). In addition, phase-amplitude correlations (PAC) indicate the modulation of faster cortical oscillations by slower oscillations in the same area (Palva and Palva, 2018, Sotero et al., 2015).

Although the effects of PA and HIE on cortical functional networks have been studied in neonates (McLaren et al., 2019, Syvälahti et al., 2024), there are no reports on long-term changes later in childhood. In this study, we investigate differences in local EEG metrics (amplitude and PAC) and cortical activity networks (AAC and PPC) among two-year-old children: those with a history of PA, those with mild to moderate HIE (HIE12), and their healthy control (HC) peers. Moreover, we correlate observed differences in these EEG metrics to neurodevelopmental scores at two years.

## 2 Methods

**Fig. 1:**
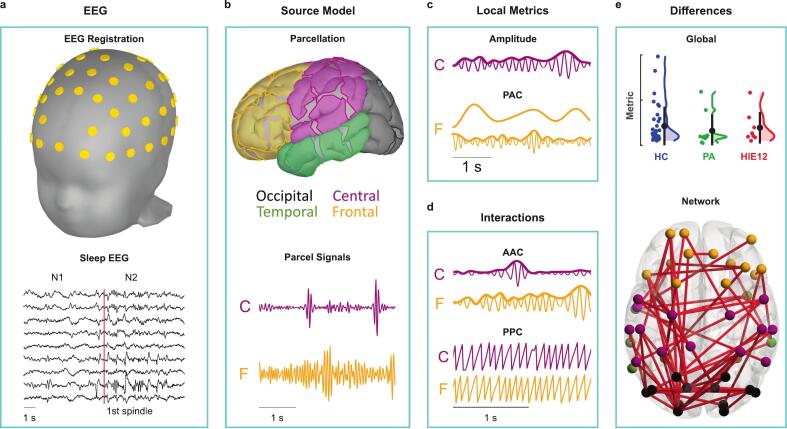
Summary of the study design. a Daytime sleep EEG recordings, which included N1 and N2 states, were collected from two-year-old children with asphyxia and their healthy peers using a 64-channel set-up. b The EEG data were pre-processed and source-reconstructed into cortical activity using a 58-parcel scheme. Each parcel was labelled according to its anatomical location and had a corresponding parcel signal. c These parcel signals were further used to compute local EEG metrics: amplitudes and phase-amplitude correlation (PAC). d Functional interactions between cortical regions were assessed using amplitude-amplitude (AAC) and phase-phase correlations (PPC). e Group differences were tested first at the global level using the mean of each metric, and then at the local level for individual electrodes/parcels or functional connections.

An overview of the methods used in this study is presented in Fig. 1**,** and further details are described in the following sections.

### 2.1 Subjects

**Fig. 2:**
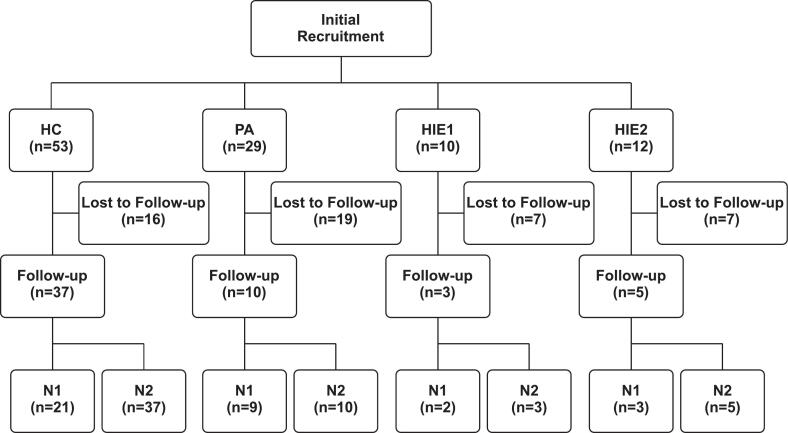
Flowchart of recruitment of subjects in this follow-up study. The initial study (Syvälahti et al., 2024) was conducted on newborns with this follow-up conducted at two years of age. HC = healthy controls, PA = perinatal asphyxia, HIE1 = mild hypoxic-ischemic encephalopathy, and HIE2 = moderate hypoxic-ischemic encephalopathy.

This study is a follow-up to initial work conducted on newborns with perinatal asphyxia, with or without mild-to-moderate HIE (Syvälahti et al., 2024). Subjects for the initial study were recruited between September 2016 and September 2020. Subjects included in this study had at least one of the following signs of asphyxia: an umbilical arterial cord pH below 7.10, a 1-min Apgar score below 6, need for assisted ventilation, and/or cardiopulmonary resuscitation at birth. Subjects with congenital anomalies, chromosomal abnormalities, infections, or other neurological conditions were excluded from this study. Further clinical description of the study subjects is presented in our previous work (Tuiskula et al., 2025). The cohort was divided into three groups: perinatal asphyxia without HIE (PA), mild to moderate HIE (HIE12), and healthy controls (HC). Clinical characteristics at birth are summarized in Table 1. EEG recordings at the age of 2.07 ± 0.10 (mean ± std) were available for ten children with PA, eight with HIE12, and 37 HC. For all the subjects, EEG recordings from N1 and N2 sleep states were collected. Fig. 2 shows the flow of recruitment of subjects for this study and the number of recordings for each group. Due to a small sample size, N1 analysis of the HIE12 group was not conducted. This study was approved by the hospital district of Helsinki and Uusimaa (HUS/1331/2016). Informed consent was obtained from the guardians of the participating children.

**Table 1:** Comparison of the clinical data for HC, PA, and HIE12 groups. Continuous data were compared with Wilcoxon rank-sum tests (alpha = 0.05, two-tailed), and categorical data was compared with Fisher's exact test (alpha = 0.05). Significant Bonferroni corrected p-values are shown in bold. a = HC vs. PA, b = HC vs. HIE12, c = PA vs. HIE12, and n.s. = nonsignificant in all comparisons. For the effect size of Wilcoxon rank-sum tests we used rank-biserial correlation and for Fisher's exact tests odds ratio, where applicable (NA = not applicable).

	HC (n = 37)	PA (n = 10)	HIE12 (n = 8)	p-values	Effect size
Sex (% male)	47.2 %	40 %	37.5 %	**n.s.**	1.3^a^, 1.5^b^, 1.1^c^
GA (weeks)	39.94 ± 0.82	40.91 ± 0.99	40.3 ± 1.44	**<0.05^a^**	0.38^a^, 0.07^b^, 0.25^c^
Birth weight (g)	3456 ± 303	3682 ± 515	3549 ± 347	**n.s.**	0.16^a^, 0.09^b^, 0.07^c^
Maternal age (a)	33.4 ± 3.9	33.2 ± 3.2	33.9 ± 2.4	**n.s.**	0.03^a^, 0.09^b^, 0.03^c^
Multipara (%)	50	20	13	**n.s.**	4^a^, 7^b^, 1.8^c^
Apgar 1 min	9.03 ± 0.29	3.89 ± 2.57	2.1 ± 1.25	**<0.001^a,b^**	0.81^a^, 0.87^b^, 0.34^c^
Apgar 5 min	9.76 ± 0.44	5.89 ± 1.83	3.5 ± 1.41	**<0.001^a,b^, <0.05^c^**	0.79^a^, 0.79^b^, 0.60^c^
Apgar 10 min	10 ± 0	7.2 ± 0.79	5.5 ± 1.31	**<0.001^a,b^, <0.05^c^**	0.90^a^, 0.91^b^, 0.62^c^
Cord arterial pH	7.29 ± 0.06	7.05 ± 0.11	7.09 ± 0.07	**<0.001^a^, <0.01^b^**	0.67^a^, 0.55^b^, 0.22^c^
Cord arterial BE	−2.56 ± 2.6	−8.19 ± 3.81	−8.2 ± 2.2	**<0.001^a^, <0.01^b^**	0.56^a^, 0.50^b^, 0^c^
Therapeutic hypothermia (%)	0	0	37.5	**<0.05^b^**	NA
Monolingual (%)	80	50	100	**n.s.**	0.24^a^
Both parents with third degree education (%)	36	30	63	**n.s.**	1.3^a^, 0.34^b^, 0.26^c^
Family history of developmental delay (%)	22	0	0	**n.s.**	NA

### 2.2 EEG recordings

We recorded the electroencephalography (EEG) signals of non-rapid eye movement (NREM) daytime sleep, which included N1 and N2 stages. The total recording times for each group were: 76.7 ± 26.8 min (mean ± std) for HC, 70.7 ± 24.1 min for PA, and 59.6 ± 14.9 min for HIE1. The subjects’ ages at recording were 2.10 ± 0.20 years (mean ± std) for the PA group, 1.99 ± 0.08 years for the HIE12 group, and for the HC group 2.07 ± 0.06 years respectively. For the recording, we used sintered Ag/AgCl electrodes with a 64-channel cap (Waveguard, by ANT GmbH, Germany) according to the 10–10 placement criteria with the recording reference placed at CPz and the ground at AFz (Fig. 1a). The EEG was recorded with an eego amplifier (ANT GmbH, Germany) at a sampling rate of Fs = 500 Hz.

### 2.3 Developmental assessment

We examined the two-year-olds using the Bayley Scales of Infant and Toddler Development 3rd Edition (BSID-III) to assess the overall development of the children (Bayley, 2006). The BSID-III provides a score on five subscales: cognitive, receptive language, expressive language, fine motor and gross motor. For each of the subscales, a standard score was acquired (normative mean = 10; score below 7 is considered significantly below average) (Bayley, 2006). All subjects in the PA and HIE12 groups had BSID-III assessments. For HC, all but two subjects had BSID-III assessments. We used the standard scores of the cognitive, receptive language and expressive language subscales to test if group differences in EEG metrics are associated with neurodevelopment.

### 2.4 EEG pre-processing

At the beginning, we located the first sleep spindle in each individual EEG, which is the transition point between N1 and N2 sleep states (De Gennaro and Ferrara, 2003). For N1 epochs we took five minutes of EEG before the spindle, and for N2 epochs we used ten min after the spindle. Subsequently, we visually identified bad channels (loose contact of the electrode, abundance of artefacts, bridging of electrodes) from the recorded data and removed them (up to six channels per subject), which resulted in the removal of an average of 2.6 channels per subject for HC, 2.1 for PA, and 1.7 for HIE12. We discarded the mastoid channels M1 and M2 from all recordings, due to insufficient quality in most recordings.

Next, the EEG data were pre-filtered within the frequency range of 0.4 Hz and 45 Hz using a pair of high-pass and low-pass Butterworth filters in forward and backward directions to avoid phase distortions and antialiasing. The stopband attenuation for the filters in one direction was at least 15 dB. The recordings were then downsampled to a sampling rate of Fs = 100 Hz to reduce data size and then converted to an average montage. We applied a semi-automatic approach (Hamaneh et al., 2014, Yrjölä et al., 2022b) to identify and remove electrocardiographic (ECG) artefacts from the data which is based on independent component analysis. Finally, for both N1 and N2 sleep states we manually selected four minutes of artifact-free EEG out of initially pre-selected epochs (of five and ten min respectively) for further analysis. The epochs were visually inspected, and sections with high amplitude spikes, technical artifacts or severe biological artifacts were rejected. We had to discard the N1 recordings from two subjects in PA, and 16 subjects in HC, as they all fell asleep too quickly. In these recordings either the first sleep spindles appeared before the 4-minute threshold of the recordings, or the epoch had too much alpha activity, which indicates that the subject had not yet transitioned into N1 sleep. The HIE12 group had to be discarded completely in N1 due to only five subjects having long enough recordings. This resulted in 21 N1 and 37 N2 recordings for the HC group, nine N1 and ten N2 recordings for PA, and eight N2 recordings for the HIE12 group. Furthermore, for the analysis we filtered all the signals into five frequency bands of interest with pairs of low-pass and high-pass Butterworth filters: low delta (0.4–1.5 Hz), high delta (1.5–4 Hz), theta (4–8 Hz), alpha (8–13 Hz), and beta (13–22 Hz). The filters used are provided in the supplementary material. Notch and mains noise were not applied due to the low frequency band of interest.

### 2.5 Computation of cortical signals

From pre-processed EEG data, we source-reconstructed the underlying cortical activity. For this, we used a realistic head model of a two-year-old toddler (O'Reilly et al., 2021) which is available in Brainstorm software (Tadel et al., 2011). The model consisted of triangulated meshes of the scalp, outer skull, and inner skull, and the cortical surface was used as the source space. It was derived from Magnetic Resonance Images (MRI) of 133 children aged 23–26 months. We downsampled the cortical mesh from the original 167,824 to 10,004 vertices to speed up computations but kept the scalp and outer/inner skull meshes at their original resolution of 2563 vertices. All sources were fixed and orthogonal relative to the cortical surface orientation. The electrode positions in the model corresponded to their placement during EEG data collection. We computed the forward operator by applying the symmetric boundary element method as it is implemented in the OpenMEEG package within Brainstorm (Kybic et al., 2005, Gramfort et al., 2010). The inverse operator was computed with dynamic statistical parametric mapping (Dale et al., 2000). We used the identity matrix as the noise covariance matrix, assuming the same level of noise in all electrodes. The following tissue conductivities were selected: 0.05 S/m for the skull, 0.33 S/m for the scalp, and 0.33 S/m for the brain. These values were interpolated from the results reported from younger and older children (Lai et al., 2005, Odabaee et al., 2014, Tokariev et al., 2016a, McCann et al., 2019). We generated a cortical parcellation scheme for this model following the procedure described in Tokariev et al. (2019b). First, we applied the k-means method to cluster all cortical sources into overall 68 bilaterally symmetric parcels (152 ± 29.8 sources per parcel). Next, we removed five pairs of symmetric parcels located in the midline of the brain. The final parcellation included 58 parcels that were assigned into four anatomical groups according to their location: occipital, frontal, central, or temporal (see Fig. 1b and Supplementary Fig. S5). This parcellation aims to achieve an optimal trade-off between spatial resolution and the reliability of cortical signal reconstruction. The complete atlas and the down-sampled cortical surface, which are readily compatible with Brainstorm software, are provided in the supplementary files (“MyAtlas.mat” and “cortex.mat”). Finally, we computed parcel signals as the weighted mean of the source signals within the parcel using weights produced by simulations (Asayesh et al., 2024, Tokariev et al., 2019b).

### 2.6 Computation of local EEG metrics

First, we analysed the mean amplitude both globally across all EEG electrodes and individually for each separate electrode (Fig. 1c). We computed the amplitudes of the Hilbert-transformed electrode signals for each of the frequency bands: 0.4–1.5 Hz (low delta), 1.5–4 Hz (high delta), 4–8 Hz (theta), 8–13 Hz (alpha), and 13–22 Hz (beta).

In addition, we computed local phase-amplitude correlation (PAC) for individual cortical parcels, which reflects temporal coordination of distinct brain rhythms within regions. PAC was computed with the nestedness coefficient, which is the phase locking value (PLV) (Lachaux et al., 1999) between the phase of the low-frequency (nesting) oscillations ($\varphi_{L}$) and the phase of the amplitude envelope of the high-frequency (nested) oscillation ($\varphi_{H}$) after filtering it with the same filter that was used for the low-frequency signal (Vanhatalo et al., 2005, Tokariev et al., 2016b). Thus, PAC is defined as

$$\mathrm{PAC}=\frac{1}{n}\left|\sum_{k=1}^{n}e^{i\left(\varphi_{L}\left(k\right)-\varphi_{H}\left(k\right)\right)}\right|.$$

We used the low delta band (0.4–1.5 Hz) as nesting frequency and theta (4–8 Hz), alpha (8–13 Hz), and beta (13–22 Hz) bands as nested frequencies.

### 2.7 Computation of functional connectivity

In addition to the local EEG measures, we computed the functional connectivity between all parcel pairs as estimations of phase–phase correlation (PPC), and amplitude–amplitude correlation (AAC) (Fig. 1d). PPC, which denotes the consistency of phase differences between signals, was estimated with the debiased weighted phase-lag index (dwPLI) (Vinck et al., 2011). We chose the dwPLI due to its robustness to the effects of volume conduction by ignoring near zero phase lags as they are likely to originate from the same source (Palva and Palva, 2012, Palva et al., 2018). The dwPLI is specified as

$$\mathrm{dwPLI}=\frac{\left|E[Im(S)]\right|}{E[|Im(S)|]},$$

where *E*[] is the expectation, S denotes the cross-spectrum of the two analysed signals, and *Im*() is the imaginary portion of the value. AAC is considered to represent the gross co-modulation between cortical regions over a time span of seconds. We estimated the AAC with the orthogonalized correlation coefficient (oCC) (Brookes et al., 2012, Hipp et al., 2012). To compute AAC we first orthogonalized each signal pair to reduce the effects of volume conduction to the correlations. Then, we applied Hilbert transforms to the orthogonalized signals to compute the amplitude envelopes of the signals. Finally, we computed the Pearson correlation coefficient between the amplitude envelopes. The oCC is defined as

$$\mathrm{oCC}=corr(x,y_{⊥x}),$$

where *corr* is the Pearson correlation coefficient between the signals and $y_{⊥x}$ is the orthogonalized signal *y* relative to *x*.

The connectivity for AAC and PPC was computed for the whole four-minute-long epochs, for both sleep states (N1 and N2) and all five frequency bands. The resulting connectivity matrices were symmetric (58 × 58) and included 1653 unique connections for each subject (Fig. 1e). In addition, we analysed the asymmetry of the resulting networks by computing the asymmetry index (AI) for each network. The AI was defined as

$$\mathrm{AI}=\frac{L-R}{L+R},$$

where *L* is the mean connectivity of the left hemisphere and *R* the mean connectivity of the right hemisphere.

### 2.8 Statistical analysis

To compare global metrics (amplitudes, AAC, PPC, and PAC) between groups, we used two-tailed Wilcoxon rank-sum tests with a significance level of α = 0.05. Pairwise comparisons were performed separately for each sleep state, and frequency band, and followed by Bonferroni correction to control for multiple comparisons (adjusted threshold: 0.05 ÷ 3 tests = 0.017). In addition, to obtain family-wise confidence intervals for the median difference across the five frequency bands, we used a permutation-based max-statistic procedure (10000 permutations, α = 0.05, studentized) (Supplementary Table S2). Local amplitudes were compared between individual electrodes using two-tailed Wilcoxon rank-sum tests. Differences between individual edges (functional connections) in AAC/PPC networks and local PAC levels (for parcels) were tested using two one-tailed Wilcoxon rank-sum tests. The subset of significantly different edges formed the contrast (or differential) network. Effect size for network differences was estimated using rank-biserial correlation. For local measures, we controlled for multiple comparisons by rejecting the fraction of significant observations with the weakest p-values (Palva et al., 2010). This fraction was computed as 5 % of the overall number of tests, leading to the rejection of the following numbers of significant observations: N = 3 for amplitudes (0.05 × 62 electrodes), N = 3 for PAC (0.05 × 58), and N = 83 for AAC/PPC networks (0.05 × 1653 edges). Moreover, to assess the statistical significance of these networks we used non-parametric inference on these patterns across frequency bands using permutation testing. We computed edgewise the Wilcoxon rank-sum z-score for each edge. Then computed a score for the pattern mean of the absolute values of the z-scores. The statistical significance was then assessed with permutation testing (10000 permutations). A p-value for each pattern was produced for each frequency band. The p-values for each pattern were then corrected across all frequency bands with the Benjamini-Hochberg method. The q-value for the pattern in its original frequency band was used as the estimate of significance for each pattern. Simultaneously, 95% confidence intervals for effect sizes for each pattern were computed using studentized max-stat. The results of non-parametric permutation testing are included in the supplement (Supplementary Table S1). To test the relationship between mean connectivity in the contrast networks and Bayley scores, we used Spearman correlation. In this work, we used statistical tests as they are implemented in MATLAB 2022b.

### 2.9 Analysis software

All preprocessing and analyses were conducted with Matlab R2022b (MathWorks, Natick, MA). We used Brainstorm (Tadel et al., 2011) and openMEEG (Gramfort et al., 2010) to create the head model. ECG artefact removal was implemented with FastICA, (https://research.ics.aalto.fi/ica/fastica/). EEGLAB (Delorme and Makeig, 2004) was used for visualization of amplitudes.

## 3 Results

### 3.1 Global amplitudes are not affected by severity of asphyxia

**Fig. 3:**
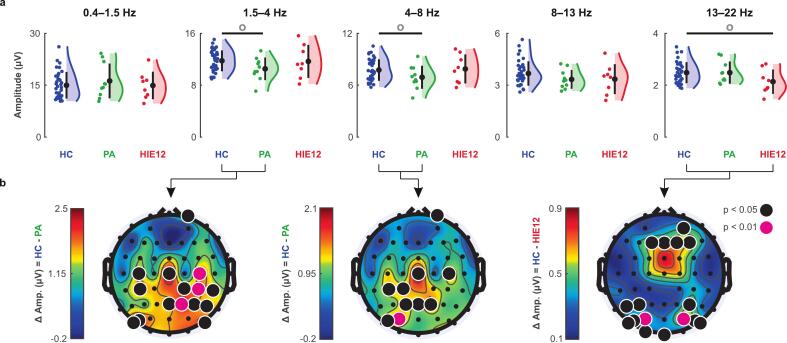
Effects of asphyxia on frequency-specific amplitudes during N2 sleep. a Differences in global mean amplitudes between healthy controls (HC, blue), perinatal asphyxia (PA, green), and hypoxic-ischemic encephalopathy (HIE12, red) groups. Coloured dots represent individual subjects from the respective groups, black dots indicate mean values, and whiskers show standard deviations. Circles on top highlight group differences with p < 0.1 (Wilcoxon rank-sum test, uncorrected). b Topographic maps show the distributions of amplitude differences (Δ) across electrodes for the cases marked with circles on a. Black and magenta circles highlight electrodes that showed statistically significant differences between groups (with p < 0.05 and p < 0.01 respectively; survived post hoc correction).

The comparison of global EEG amplitudes between groups did not reveal any significant differences during neither N1 nor N2 sleep. However, we found three nearly significant (uncorrected p < 0.1) cases during N2 stage (Fig. 3a). In the neighbouring high delta (1.5–4 Hz) and theta (4–8 Hz) frequency bands two-year-old children with PA showed lower levels of global amplitudes relative to controls (HC > PA; uncorrected p = 0.071, Bonferroni p = 0.21, q-FWE (family-wise error) = 0.24 and uncorrected p = 0.094, Bonferroni p = 0.27, q-FWE = 0.32 respectively, Wilcoxon test). At beta frequencies (13–22 Hz), the HIE12 group had decreased amplitudes compared to controls (HC > HIE12; p = 0.052, Bonferroni p = 0.15, q-FWE 0.17, Wilcoxon test). Further, for these cases we also compared amplitudes between individual EEG electrodes (Fig. 3b). We found that the PA group exhibited lower amplitudes in the 1.5–8 Hz frequency range, primarily at parietal electrodes. In contrast, HIE12 toddlers showed attenuated amplitudes at beta frequencies (13–22 Hz) in two clusters of electrodes: frontal and occipital.

### 3.2 Asphyxia associates with attenuated connectivity in broad AAC networks

In N1 sleep, we did not find any significant differences between PA and HC groups in global AAC (Fig. S1**a**). Nevertheless, we found significant differences in individual functional connections between parcels, which resulted in contrast networks. In the low delta frequency band (0.4–1.5 Hz), the PA group showed stronger connectivity in K ≈ 6% of edges, which were mostly located in the right hemisphere. In the rest of the frequency bands (except alpha), two-year-old children with PA had attenuated connectivity with the most prominent effect observed in the theta band (4–8 Hz; HC > PA, K = 21%). The theta contrast network was also primarily lateralized to the right hemisphere.

**Fig. 4:**
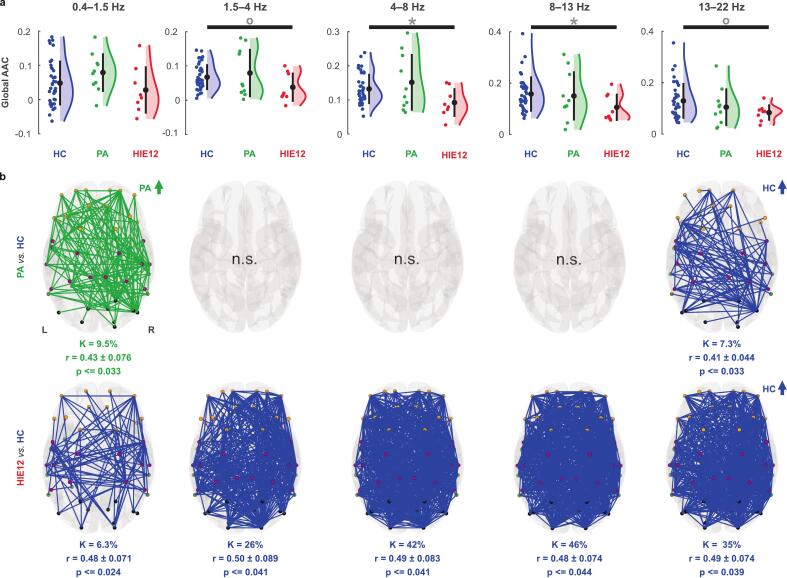
Effects of asphyxia on amplitude-amplitude correlations (AAC) during N2 sleep. a Comparisons of global AAC levels between groups: healthy controls (HC, blue), children after perinatal asphyxia (PA, green), and with hypoxic-ischemic encephalopathy (HIE12, red). Coloured dots show individual subjects, black dots stand for group means, while whiskers indicate standard deviations. Significance levels (Wilcoxon rank-sum test) are marked as follows: a circle for p < 0.1, an asterisk for p ≤ 0.05; grey colour denotes the cases that did not survive Bonferroni correction. b Glass brains show group differences in AAC cortical networks (Wilcoxon rank-sum test; corrected for multiple comparisons): PA vs. HC (top) and HIE12 vs. HC (bottom). Colour of the edge's highlights in which group AAC was stronger. Network density (K) reflects the percentage of edges in the contrast network compared to the full network. Effect size for the contrast networks was estimated with rank-biserial correlation (r). Nodes are colour coded according to their anatomical locations: frontal (orange), central (magenta), temporal (green), and occipital (black).

During N2 sleep, the HIE12 group had lower global AAC connectivity compared to HC in the theta-alpha frequency range (4–13 Hz; HC > HIE12, uncorrected p ≤ 0.034, Bonferroni p ≥ 0.087 for both; see Fig. 4a). Correction for family-wise error resulted in a significant difference in theta band (q-FWE = 0.039) and not significant in alpha band (q-FWE = 0.17). Neighbouring high delta (1.5–4 Hz) and beta (13–22 Hz) bands showed similar group differences, but the statistics were slightly above the significance threshold (0.05 < uncorrected p < 0.1). However, all these cases of global differences did not pass post hoc Bonferroni correction (Bonferroni p > 0.05). At the level of individual edges, the PA group showed elevated connectivity in the low delta band (HC < PA; 0.4–1.5 Hz; Fig. 4b**,** top), similar to the pattern observed during N1 sleep. In turn, at the highest frequencies, PA showed reduced connectivity in K ≈ 7% of the network affecting mostly interhemispheric connections (13–22 Hz; HC > PA). Notably, the HIE12 subjects showed attenuated connectivity across all frequencies in widespread cortical networks compared to HC (Fig. 4b, bottom). The strongest effect was seen at the theta-alpha frequencies, where the contrast networks comprised K = 42–46% from all connections. Finally, we tested whether group differences in global amplitudes (all p < 0.1 cases in Fig. 3a) could potentially drive AAC differences. However, we found no significant correlations (Spearman test) between amplitudes and global AAC levels (Fig. S2, top).

### 3.3 PA and HIE show opposite effects in constrained PPC networks

For global PPC strength during N1, we found a significant difference between the HC and PA groups only at high delta frequencies (1.5–4 Hz; HC > PA, uncorrected p = 0.046, Bonferroni p = 0.092), though this did not pass the Bonferroni correction (Fig.S1**b,** top). At the level of individual edges, the PA group showed stronger connectivity in K = 10% the of the whole network at low delta (0.4–1.5 Hz). In other frequency bands (except theta), PA subjects exhibited reduced connectivity within sparse (K = 1.3–4.7%) and uniformly distributed patterns (Fig.S1**b,** bottom).

**Fig. 5:**
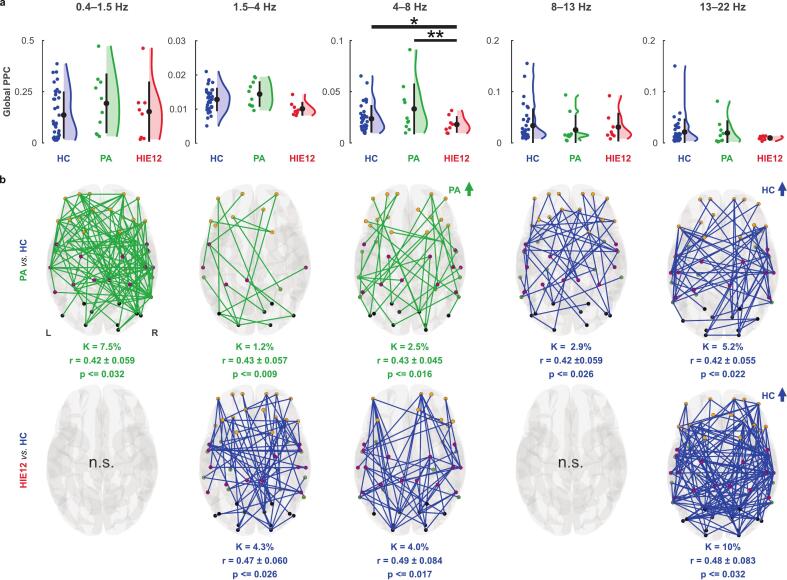
Effects of asphyxia on phase-phase correlations (PPC) during N2 sleep. a Group differences in the global levels of PPC strength between healthy controls (HC, blue), toddlers after perinatal asphyxia (PA, green), and toddlers with hypoxic-ischemic encephalopathy (HIE12, red). Coloured dots represent individuals from the corresponding groups, black dots show group means, and whiskers indicate standard deviations. The significance levels (Wilcoxon rank-sum test) are shown as follows: * p ≤ 0.05, and ** p ≤ 0.01. Grey colour of asterisks indicates cases that did not pass Bonferroni correction. b Glass brains show edges that were significantly different between groups (Wilcoxon rank-sum test, corrected): PA vs. HC (top), and HIE12 vs. HC (bottom). Colours of edges indicate the group, where connectivity was stronger. Network density (K) represents the proportion of edges that differ between groups. Effect size for contrast networks was assessed using rank-biserial correlation (r). Nodes are colour coded according to their anatomical locations: frontal (orange), central (magenta), temporal (green), and occipital (black).

During N2 state, global PPC level was reduced in HIE12 relative to controls (HC > HIE12; p = 0.025) and two-year-old children with PA (PA > HIE12; uncorrected p = 0.009, Bonferroni p = 0.027, q-FWE = 0.74) in high delta band (1.5–4 Hz; Fig. 5a). However, edge-by-edge comparison revealed the most prominent effect for the PA group in the low delta band (0.4–1.5 Hz), where K ≈ 8% of network showed increased PPC (HC < PA; Fig. 5b, top). The PA group also had higher PPC connectivity in the neighbouring frequencies (1.5–8 Hz; HC < PA; K ≈ 1–3%), but reduced connectivity at higher frequencies (8–22 Hz; HC > PA; K ≈ 3–5%), though all these patterns were relatively sparse. In contrast to PA, the HIE12 group exhibited reduced connectivity (HC > HIE12; Fig. 5b, bottom) at mid frequencies (1.5–8 Hz; K ≈ 4%). Similarly to PA, at the highest frequencies (13–22 Hz; K = 10%) HIE12 showed weaker connectivity to controls in a broader pattern. Analogously to AAC, we did not find any significant correlations between global levels of amplitudes and PPC connectivity (Fig. S2, bottom).

### 3.4 PAC is reduced in PA

**Fig. 6:**
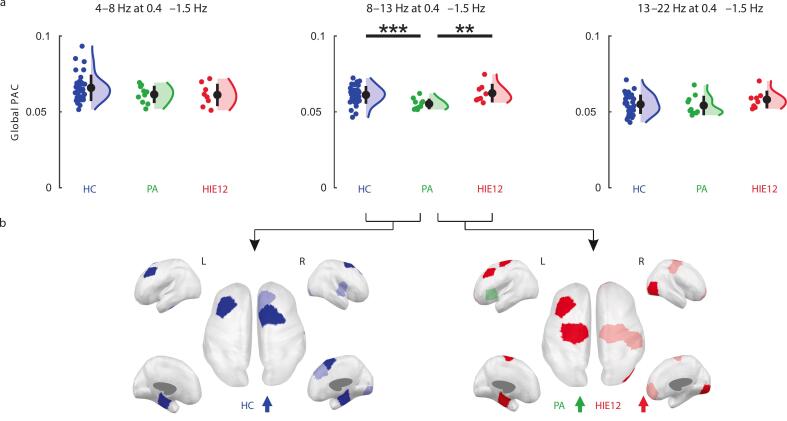
Effects of asphyxia on phase-amplitude correlation (PAC) during N2 sleep. a Comparison of global PAC between study groups: healthy controls (HC, blue), toddlers after perinatal asphyxia (PA, green), and toddlers diagnosed with hypoxic-ischemic encephalopathy at birth (HIE12, red). Coloured dots represent subjects from each group, black dots indicate group means, and whiskers show standard deviations. The significance levels (Wilcoxon rank-sum test) are shown as follows: ** p ≤ 0.01, and *** p ≤ 0.001. Low delta (0.4–1.5 Hz) was used as the nesting frequency, while theta (4–8 Hz), alpha (8–13 Hz), and beta (13–22 Hz) served as the nested frequencies. b Parcels with group differences in local PAC are highlighted. Colours show which group had stronger PAC (Wilcoxon rank-sum test). Pale colours highlight parcels which were rejected at post hoc correction for multiple comparisons.

Comparing phase-amplitude correlation (PAC) during N2 sleep between groups revealed reduced global PAC in two-year-old children with PA compared to both HC (HC > PA; p < 0.001, Bonferroni p < 0.001, q-FWE < 0.01) and HIE12 (PA < HIE12; p = 0.012, Bonferroni p = 0.036, q-FWE = 0.15) when alpha (8–13 Hz) was nested on low delta (1.5–4 Hz) frequencies (Fig. 6a). Further, we compared local PAC between corresponding parcels for these groups (Fig. 6b). Compared to HC, the PA group showed significantly reduced PAC in a pair of bilateral central and a pair of medial temporal cortices (HC > PA; uncorrected p ≤ 0.022, all passed post hoc correction). A few parcels in the right hemisphere (frontal, central and occipital) also showed lower PAC in the PA group; however, p-values (0.033 < uncorrected p < 0.047) did not pass correction. A spatially similar pattern of parcels in PA exhibited attenuated PAC relative to HIE12. However, more significant differences showed parcels of the left hemisphere (PA < HIE12; uncorrected p < 0.022, passed correction). During N1 sleep, PA toddlers also showed lower global PAC compared to HC for nested alpha frequencies, but the statistical significance was slightly above the threshold (HC > PA; uncorrected p < 0.094; Fig.S3).

### 3.5 Asphyxia-related network effects show only variable links to the concurrent cognitive performance

In search of further clinical correlates, we compared the asphyxia-related network effects at two years of age to the subjects' concurrent cognitive and language performance assessed with standard scores of BSID-III subscales. We found no correlations between receptive or expressive language subscale scores and contrast (differential) networks. No consistent relationships were observed in the correlations between mean network connectivity levels and cognitive subscale scores. Only three out of the 23 AAC and PPC networks studied showed statistically significant correlations (Fig. S4), but they were from different coupling modes (AAC vs PPC), sleep state (N1 vs N2) and frequency band (delta or alpha). Moreover, the direction of correlation appeared to differ between subjects in the HC and asphyxia groups. Taken together, the observations do not support a robust link between computational measures of cortical activity networks and subjects’ assessable cognitive and language performance at two years of age. In addition, we correlated the AI of each network to the BSID-III subscales but found no correlations between them.

## 4 Discussion

Our study shows that perinatal asphyxia with or without HIE has an effect on cortical activity that is still observable at two years of age. Infants' perinatal asphyxia showed a clearly graded effect on cortical activity at term age (Syvälahti et al., 2024), but, surprisingly, at two years, we found a deviation in the activity networks between PA and HIE12 which may indicate a divergence in cortical development.

We found no statistically significant differences in global EEG amplitudes between the groups (Fig. 3). However, analysis between individual electrodes revealed spatial- and frequency-specific differences between PA and HC as well as between HIE12 and HC. Remarkably, these findings differ from those reported in infants, where a graded response in global amplitudes as a function of HIE severity was found. However, the infants showed no significant difference between control group and PA in either global or single channel amplitudes (Syvälahti et al., 2024).

Previous research linked AAC and PPC networks to higher cognitive functions in adults (Colonnese et al., 2010, Palva et al., 2018, Syvälahti et al., 2024, Tokariev et al., 2016b, Tuiskula et al., 2025, Vinck et al., 2011) and demonstrated their relation to neurodevelopment in infants (Tokariev et al., 2019a, Yrjölä et al., 2022a). Although group differences in global connectivity were not pronounced, we found robust differences in network patterns across most of the frequencies comprising up to half of the connections (specifically in HIE12 AAC networks). Effects of PA on PPC networks were spatially more constrained. Interestingly, no PPC differences were found between any of these groups at term age (Syvälahti et al., 2024). The current findings may indicate that the effects of perinatal asphyxia on PPC networks become apparent only later in development. Moreover, our work shows clear differences in both AAC and PPC connectivity between PA and HC. Despite intuitive expectations, our findings suggest that these differences are not expressed as a graded response to the severity of asphyxia. We found opposite effects for PA and HIE12 as compared to controls. This was evident as for both AAC and PPC, we found stronger connectivity in the PA group compared to the HC group for lower frequency bands (0.4–8 Hz), whereas HIE12 had reduced connectivity compared to controls in all frequency bands. In contrast, in our previous work on infants (Syvälahti et al., 2024) we did not find any differences between controls versus PA or mild HIE groups during quiet sleep (precursor of non-REM sleep, analysed here (Brookes et al., 2012, Palva et al., 2010)) for AAC. At the same time, infants with moderate HIE demonstrated stronger connectivity in large-scale AAC networks across all frequencies (Syvälahti et al., 2024).

Lastly, we investigated the effects of perinatal asphyxia on PAC, which has been characterized as a mechanism to transfer information between networks on different spatiotemporal scales. Here, we found only one instance of a significant difference in PAC between the groups, where PA had a significantly lower PAC compared to both HC and HIE12 in low delta band nesting alpha band during N2 sleep. In contrast, this finding is different from the PAC results as infants, where a clearly graded response was found (Syvälahti et al., 2024).

It should be noted that the cortical activity networks at two years did not show clear correlations to concurrent cognitive performance. Prior work has reported correlations from the neonatal cortical networks to later neurocognition (Tokariev et al., 2019a, Al-Sa'd et al., 2024, Nghiem et al., 2019, Alcauter et al., 2015). However, neonatal cortical activity and its networking are based on different neuronal mechanisms compared to the cortical activity at 2 years (Luhmann et al., 2022, Molnár et al., 2020, Vanhatalo and Kaila, 2006). The prior studies therefore demonstrate the infant brain's potential to develop later cognitive performance, which is not conflicting with the present findings on concurrent correlations. Furthermore, our current findings suggest opposite directions of correlations in the healthy control subjects compared to the two-year-old children with asphyxia, which is fully in line with prior studies that jointly suggest that developmental adversities (e.g. drug exposure, prematurity, asphyxia) may disrupt the links between network strengths and cognitive performance (Tokariev et al., 2022, Tokariev et al., 2019b, McLaren et al., 2019).

However, it should be noted that our study has some limitations. Subject recruitment and participation in follow-up visits were affected by the restrictions of the covid-19 pandemic. Moreover, it was difficult to obtain enough data from N1 sleep as most of the subjects transitioned rapidly to deeper sleep, which resulted in not enough data for analysis. This reduction in N1 recordings might have influenced the results for this state and should be considered when interpreting them. Altogether, this resulted in the need to combine the mild and moderate HIE groups together and discarding the N1 analysis for the HIE groups completely to preserve statistical power and maintain a meaningful contrast with the less severe PA group without encephalopathy. Consequently, the HIE-related findings should be interpreted with caution, as merging mild and moderate cases may obscure severity-specific effects. In addition, we used basic Bayley-III scores to explore whether the connectivity changes were related to early cognitive performance. However, our analyses lacked detailed background information related to language and social-cognitive development. As a result, these preliminary correlations should be interpreted with caution and the direct relationship between cortical connectivity alterations and language outcomes at two years remains to be demonstrated using richer behavioural phenotyping and larger, longitudinal cohorts. Finally, we restricted our analyses to simple bivariate statistical models to minimize overfitting and preserve interpretability due to small sample sizes. More complex multivariable models should be explored in larger cohorts. Thus, further validation is needed in interpretation of our results. Nevertheless, our work also demonstrates notable strengths. We conducted a systematically thorough analysis of multiple EEG- and connectivity-based metrics for a rarely studied cohort of two-year-old children with PA and mild to moderate HIE. Moreover, our analyses are based on realistic modelling of the head, and reconstruction of sources on the cortical surface.

In conclusion, to our knowledge this is the first study to present how perinatal asphyxia affects EEG activity networks later in childhood. Unexpectedly, even perinatal asphyxia without HIE may lead to long-lasting changes that deviate from changes after mild-to-moderate HIE. Because of the small cohort and imbalanced groups due to COVID-19 related disruptions to data collection, these preliminary findings should be interpreted with caution. Replication studies with larger cohorts comprising two-year-olds after perinatal asphyxia with and without HIE are needed to validate our current findings.

## CRediT authorship contribution statement

**Sebastian König:** Writing – original draft, Visualization, Formal analysis, Conceptualization. **Anna Tuiskula:** Writing – review & editing, Resources, Investigation, Data curation. **Marjo Metsäranta:** Writing – review & editing, Resources, Investigation, Data curation. **Susanna Stjerna:** Investigation. **Emma Saure:** Investigation. **Leena Haataja:** Writing – review & editing, Resources, Investigation, Data curation. **Sampsa Vanhatalo:** Writing – review & editing. **Anton Tokariev:** Writing – original draft, Methodology, Conceptualization.

## Declaration of Competing Interest

The authors declare that they have no known competing financial interests or personal relationships that could have appeared to influence the work reported in this paper.

## Data Availability

MATLAB code for source-level connectivity analysis, frequency-specific connectivity matrices used in this study, and other derivatives of the original data have been included in the supplement where possible. Original clinical EEG data and neurocognitive performance assessments are available after making a data sharing agreement with Helsinki University Hospital.
