# Supplementary material for: Effects of perinatal asphyxia on cortical activity in two-year-old children
Source: Neuroimage Clin. 2025 Dec 17;49:103933. doi: 10.1016/j.nicl.2025.103933 (PMC12811685; doi:10.1016/j.nicl.2025.103933)
Supplement: Supplementary Data 1 [file mmc1.docx]

**Supplement**


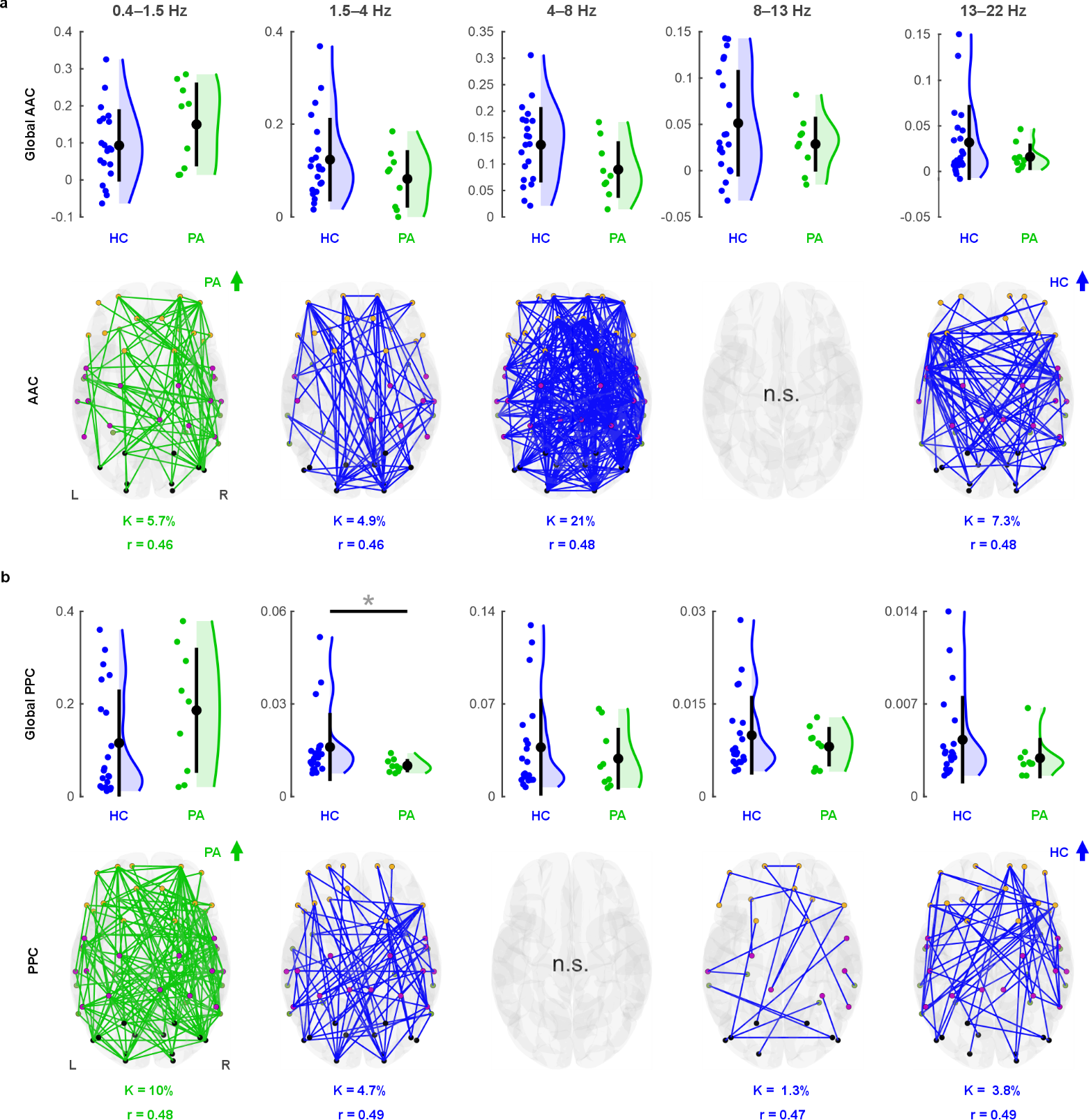


***Figure S1. Effects of asphyxia on large-scale AAC and PPC networks during N1 sleep.*** *Global cortical activity and networks for* ***a*** *AAC and* ***b*** *PPC during N1 sleep for healthy controls (HC, blue) and children with perinatal asphyxia (PA, green). Grey asterisk indicates a statistically significant group difference (p<0.05) which did not pass Bonferroni correction. Each node in the network corresponds to a cortical parcel and is color-coded according to its anatomical location (orange = frontal, magenta = central, green = temporal, and black = occipital). Edges represent connections that are statistically (two one-tailed tests, Wilcoxon rank-sum) significantly stronger in one group compared to the other. The network density, which is the proportion of edges that significant from all possible edges, is indicated as K and the effect size of the networks is estimated with the rank biserial correlation (r).*


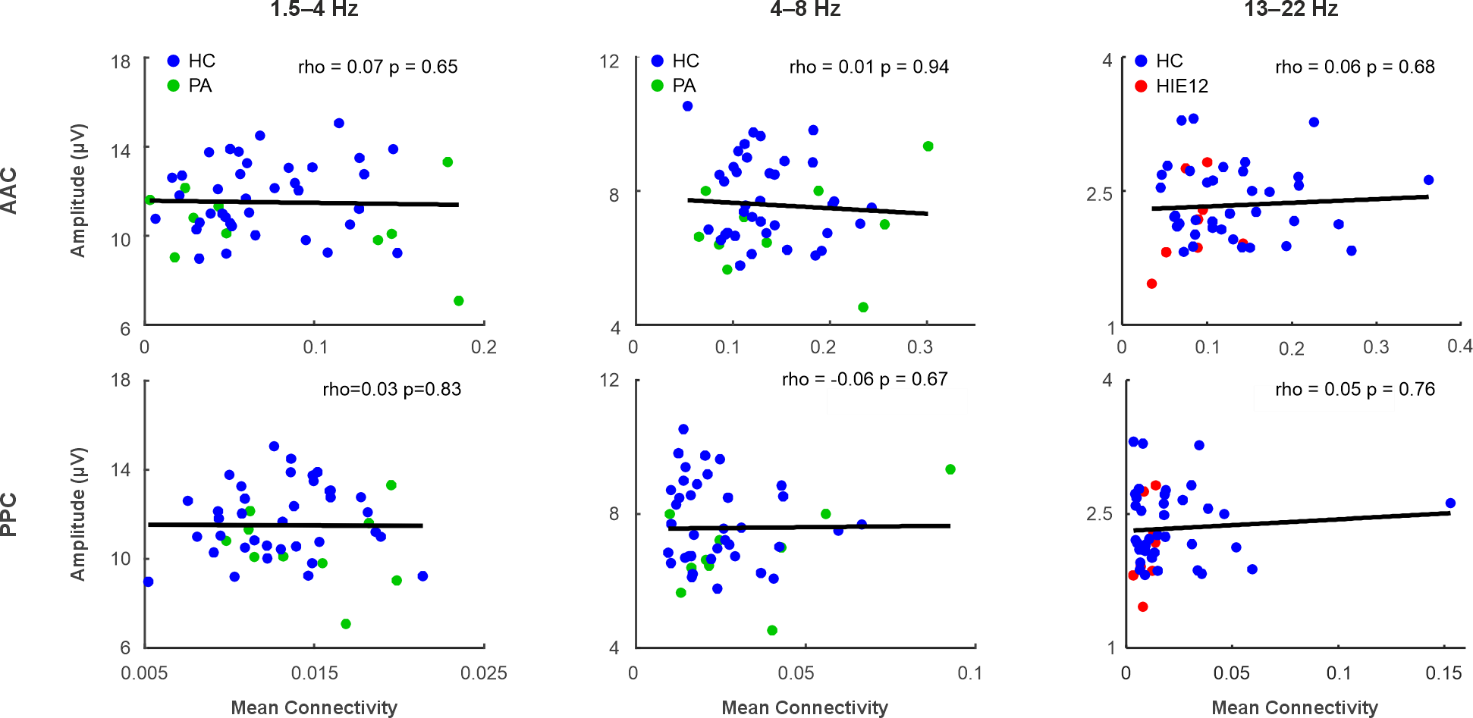


***Figure S2. Correlation between connectivity measures (AAC and PPC) and global amplitudes.*** *Correlations for the frequency bands in high delta, theta, and beta highlighted in* ***Figure 3****. Rho indicates the Spearman correlation coefficient. Black line shows the least squares fit for both groups combined.*


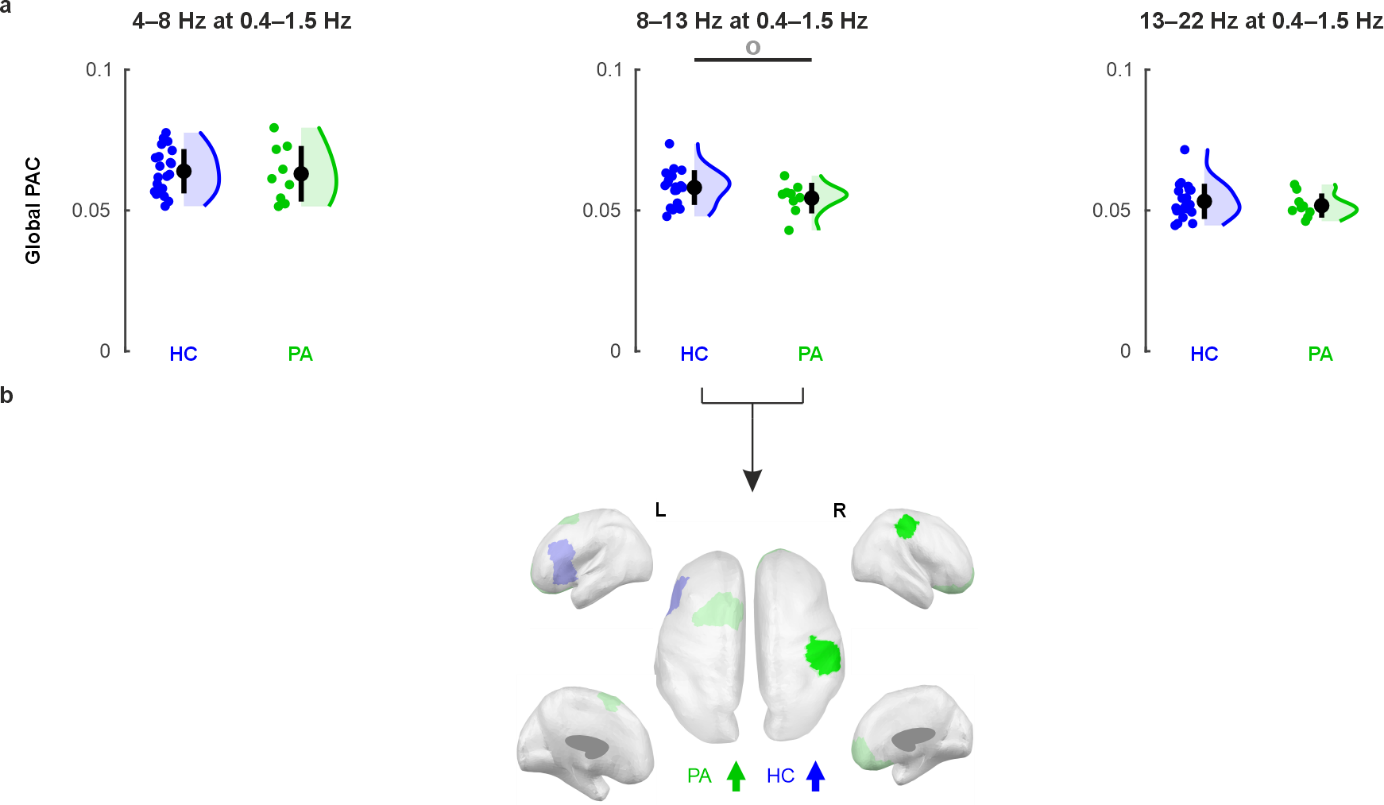


***Figure S3. Effects of asphyxia on phase-amplitude coupling (PAC) during N1 sleep. a*** *Global PAC of the study groups (green = PA for perinatal asphyxia, and blue = HC for healthy controls) with low delta frequency band (0.4 – 1.5 Hz) as nesting frequency and theta (4 – 8 Hz), alpha (8 – 13 Hz), and beta (13 – 22 Hz) frequency bands as nested frequencies. The grey circle indicates that the p-value for the group difference is close to being significant (0.05<p<0.1).* ***b*** *Differences in PAC on parcel level between PA and HC. Dark green parcels indicate a significantly higher PAC in PA compared to HC. Lighter coloured parcels (blue/green) indicate parcel differences that did not pass correction for multiple comparisons.*


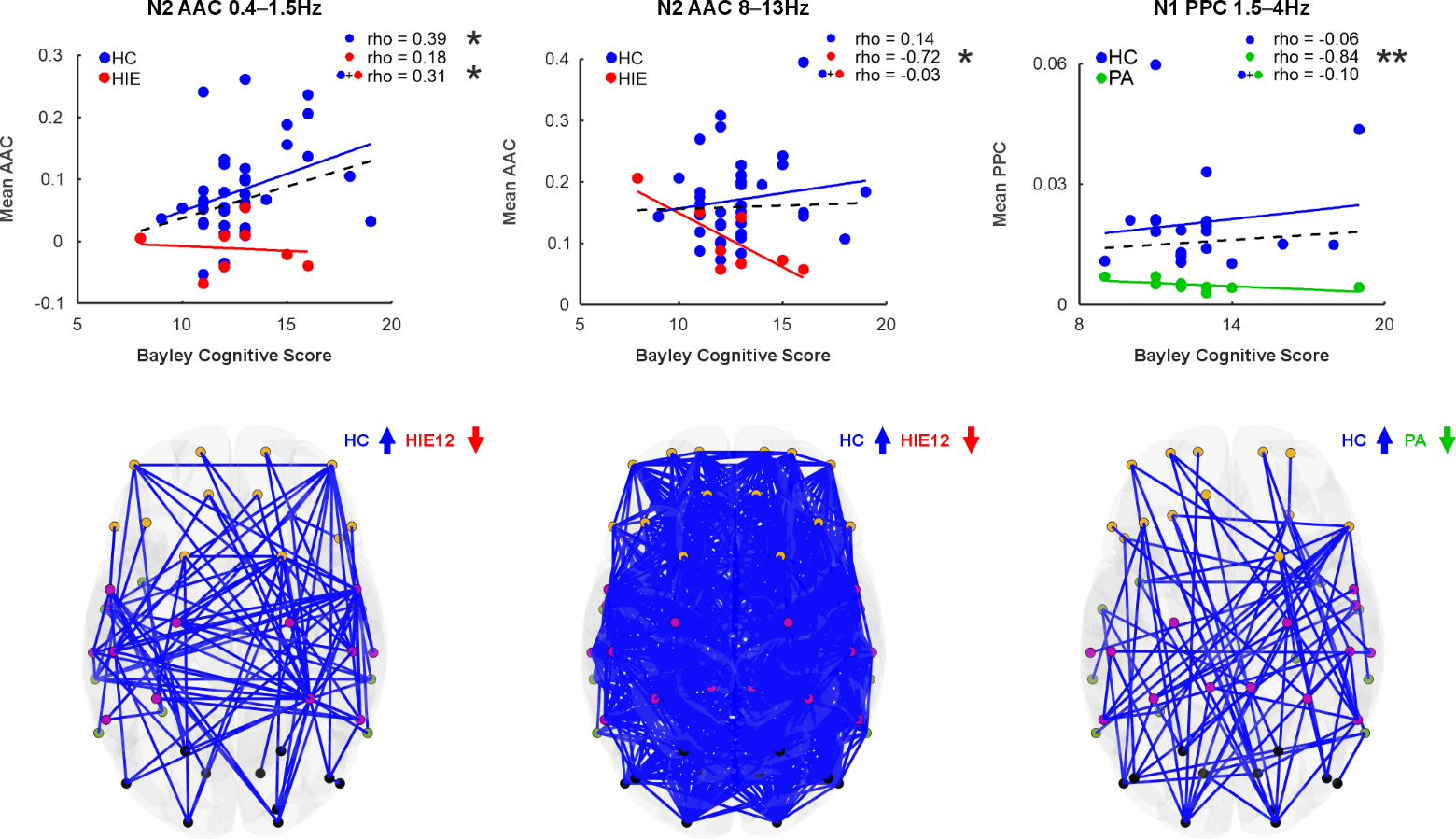


***Figure S4. Contrast networks that correlate with Bayley cognitive score.*** *Rho denotes the Spearman correlation coefficient. One asterisk indicates an FDR corrected p-value of below 0.05 for the given correlation and two asterisks indicate an FDR corrected-value of below 0.01. Lines represent the least squares fit for the given group (blue = HC for healthy controls, green = PA for children with perinatal asphyxia, and red = HIE12 for hypoxic-ischemic encephalopathy). Black dashed line shows the least squares fit for the combination of both groups.*


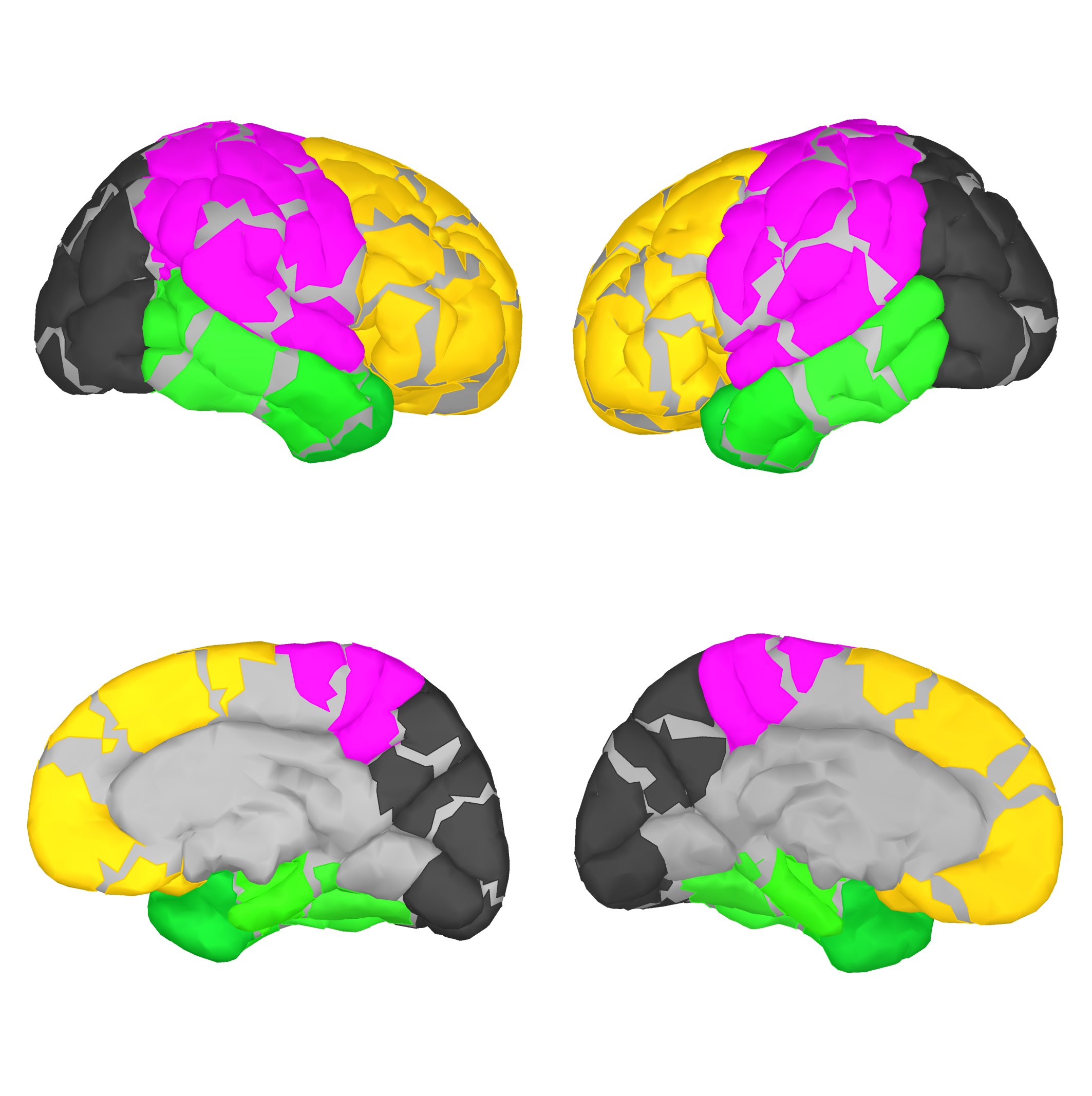
***Figure S5. Parcellation of the cortex.*** *Location of each parcel on the cortex. In total 58 parcels were used for the model. Each parcel was labelled according to its location into four groups: frontal (yellow), central (magenta), occipital (black), and temporal (green).*

| *AAC PA-HC* | *p* | *q* | *Effect* | *CI-95%* | *No. of edges* |
| --- | --- | --- | --- | --- | --- |
| *0.4 – 1.5Hz* | <0.001 | <0.001 | 0.097 | 0.036 *–* 0.16 | 156 |
| *13 – 22Hz* | 0.011 | 0.022 | 0.06 | 0.001 *–* 0.12 | 120 |
| *AAC HIE12-HC* |  |  |  |  |  |
| *0.4 – 1.5Hz* | <0.001 | <0.001 | 0.095 | 0.029 *–* 0.16 | 103 |
| *1.5 – 4Hz* | <0.001 | <0.001 | 0.057 | 0.018 *–* 0.95 | 429 |
| *4 – 8Hz* | 0.0018 | 0.0080 | 0.059 | 0.017 *–* 0.10 | 692 |
| *8 – 13Hz* | 0.0051 | 0.021 | 0.066 | 0.011 *–* 0.12 | 763 |
| *13 – 22Hz* | 0.0030 | 0.020 | 0.063 | 0.010 *–* 0.12 | 574 |
| *PPC PA-HC* |  |  |  |  |  |
| *0.4 – 1.5Hz* | <0.001 | <0.001 | 0.16 | 0.052 *–* 0.26 | 123 |
| *1.5 – 4Hz* | <0.001 | <0.001 | 0.018 | 0.0093 *–* 0.026 | 19 |
| *4 – 8Hz* | <0.001 | <0.001 | 0.022 | 0.0081 *–* 0.036 | 41 |
| *8 – 13Hz* | <0.001 | <0.001 | 0.027 | -0.0083 *–* 0.063 | 86 |
| *13 – 22Hz* | <0.001 | <0.001 | 0.016 | -0.0035 *–* 0.035 | 47 |
| *PPC HIE12-HC* |  |  |  |  |  |
| *1.5 – 4Hz* | <0.001 | <0.001 | 0.014 | 0.0070 *–* 0.022 | 71 |
| *4 – 8Hz* | <0.001 | <0.001 | 0.040 | 0.0087 *–* 0.071 | 66 |
| *13 – 22Hz* | <0.001 | <0.001 | 0.020 | -0.0010 *–* 0.042 | 171 |

***Table S1. Permutation based FDR correction over frequency bands for network patterns.*** *Non-parametric inference on the patterns presented in the study across frequency bands using permutation testing. The table shows for each pattern the p-value for the permutation, the corrected Benjamini-Hochberg q-values across the frequency bands, effect size as the mean difference between the groups in the pattern, and the confidence interval (CI) for the effect.*

| *Amplitude PA-HC* | *p* | *q* | *Effect* | *CI-95%* |
| --- | --- | --- | --- | --- |
| *1.5 – 4Hz* | 0.071 | 0.24 | 1.1 | -0.37 *–* 2.6 |
| *4 – 8Hz* | 0.094 | 0.32 | 0.7 | -0.35 *–* 1.8 |
| *Amplitude HIE12-HC* |  |  |  |  |
| *13 – 22Hz* | 0.052 | 0.17 | 0.34 | -0.089 *–* 0.77 |
| *Global AAC HIE12-HC* |  |  |  |  |
| *1.5 – 4Hz* | 0.059 | 0.075 | 0.034 | -0.0025 *–* 0.070 |
| *4 – 8Hz* | 0.029 | 0.039 | 0.038 | 0.0014 *–* 0.074 |
| *8 – 13Hz* | 0.034 | 0.17 | 0.045 | -0.012 *–* 0.10 |
| *13 – 22Hz* | 0.099 | 0.42 | 0.031 | -0.020 *–* 0.083 |
| *Global PPC PA-HC* |  |  |  |  |
| *4 – 8Hz* | 0.009 | 0.74 | 0.0044 | -0.0078 *–* 0.017 |
| *Global PPC HIE12-HC* |  |  |  |  |
| *4 – 8Hz* | 0.025 | 0.75 | 0.0031 | -0.0060 *–* 0.012 |
| *Global PAC PA-HC* |  |  |  |  |
| *8 – 13Hz* | <0.001 | 0.0052 | 0.0063 | 0.0014 *–* 0.011 |
| *Global PAC HIE12-PA* |  |  |  |  |
| *8 – 13Hz* | 0.012 | 0.15 | 0.0060 | -0.0009 *–* 0.013 |

***Table S2. Permutation based family-wise error correction for global amplitude, AAC, PPC, and PAC.*** *Family-wise confidence intervals for the median difference across the five frequency bands was computed with a permutation-based max-statistic procedure (10000 permutations, α = 0.05, studentized). The table shows for each metric with significant or close to significant group difference (p<0.1) the raw p-value, the corrected q-values across the frequency bands, effect size as the median difference between the groups in the pattern, and the confidence interval (CI) for the effect.*
