# Supplementary material for: Effects of perinatal asphyxia on cortical activity in two-year-old children
Source: Neuroimage Clin. 2025 Dec 17;49:103933. doi: 10.1016/j.nicl.2025.103933 (PMC12811685; doi:10.1016/j.nicl.2025.103933)
Supplement: Supplementary Data 2 [file mmc2.docx]

# Supplementary Code for “Effects of perinatal asphyxia on cortical activity in two-year-old children”

This repository contains the code used for analyses and figures in the manuscript. In addition, anonymized connectivity matrices for participants of the study are provided.

Files

- “5_band_filters.mat” – contains the filters used for the frequency bands of interest: low delta, high delta, theta, alpha and beta.

- “CollapseOperator.mat” – the computed weights needed to form parcel signals (see Tokariev, et al. 2019)

- "Compute_Connectivity.m” – generates a dummy EEG signal and then computes the connectivity metrics analyzed in the study: AAC, PPC, and PAC.

- “Compute_Group_Difference.m” - generates a dummy connectivity matrix for two groups and computes the group difference between them.

- “cortex.mat” - contains the downsampled cortical surface file of the two-year-old O’reilly dataset available in brainstorm software.

- “FidelityOperator.mat” - results of fidelity testing (see Tokariev, et al. 2019). This was not implemented in this study.

- “Headmodel.mat” - contains the head model used in the study.

- “InverseOperator” - contains the inverse operator of the headmodel.

- “MyAtlas.mat” - contains the parcellation used in the study.

- ”plot3D.m” - helper function used to visualize networks.

- “PreprocessEEG.m” - creates dummy EEG data and then applies the preprocessing conducted in the study.

- “tess_cortex_pial_low.mat” - variable for visualization by the “plot3D.m” function

Folders

- “Connectivity matrices” - contains the connectivity matrices of participants in the study (HC – healthy control, HIE – mild to moderate hypoxic ischemic encephalopathy, and PA – perinatal asphyxia without HIE)

Tokariev A, Stjerna S, Lano A, Metsäranta M, Palva JM, Vanhatalo S. Preterm Birth Changes Networks of Newborn Cortical Activity. Cereb Cortex. 2019 Feb 1;29(2):814-826. doi: 10.1093/cercor/bhy012. Erratum in: Cereb Cortex. 2019 Apr 1;29(4):1697. doi: 10.1093/cercor/bhy100. PMID: 30321291

Dependencies: MATLAB R2022b
